# Demand for abortion and post abortion care in Ibadan, Nigeria

**DOI:** 10.1186/s13561-014-0003-9

**Published:** 2014-06-17

**Authors:** Bosede O Awoyemi, Jacob Novignon

**Affiliations:** 1 Department of Economics, Afe Babalola University, Ado-Ekiti, Nigeria; 2 Department of Economics, University of Ibadan, Ibadan, Nigeria

**Keywords:** Abortion, Post abortion care, Nigeria, Probit

## Abstract

**Background:**

While induced abortion is considered to be illegal and socially unacceptable in Nigeria, it is still practiced by many women in the country. Poor family planning and unsafe abortion practices have daunting effects on maternal health. For instance, Nigeria is on the verge of not meeting the Millennium development goals on maternal health due to high maternal mortality ratio, estimated to be about 630 maternal deaths per 100,000 live births. Recent evidences have shown that a major factor in this trend is the high incidence of abortion in the country. The objective of this paper is, therefore, to investigate the factors determining the demand for abortion and post-abortion care in Ibadan city of Nigeria.

**Methods:**

The study employed data from a hospital-based/exploratory survey carried out between March to September 2010. Closed ended questionnaires were administered to a sample of 384 women of reproductive age from three hospitals within the Ibadan metropolis in South West Nigeria. However, only 308 valid responses were received and analysed. A probit model was fitted to determine the socioeconomic factors that influence demand for abortion and post-abortion care.

**Results:**

The results showed that 62% of respondents demanded for abortion while 52.3% of those that demanded for abortion received post-abortion care. The findings again showed that income was a significant determinant of abortion and post-abortion care demand. Women with higher income were more likely to demand abortion and post-abortion care. Married women were found to be less likely to demand for abortion and post-abortion care. Older women were significantly less likely to demand for abortion and post-abortion care. Mothers’ education was only statistically significant in determining abortion demand but not post-abortion care demand.

**Conclusion:**

The findings suggest that while abortion is illegal in Nigeria, some women in the Ibadan city do abort unwanted pregnancies. The consequence of this in the absence of proper post-abortion care is daunting. There is the need for policymakers to intensify public education against indiscriminate abortion and to reduce unwanted pregnancies. In effect, there is need for effective alternative family planning methods. This is likely to reduce the demand for abortion. Further, with income found as a major constraint, post abortion services should be made accessible to both the rich and poor alike so as to prevent unnecessary maternal deaths as a result of abortion related complications.

## Background

The demand for abortion and post abortion care continues to be an issue in both economic and political spheres. In most African countries, abortion remains both unauthorized and unsafe which is why it is the leading cause of maternal deaths accounting for a global average of 13% of fatalities related to pregnancy [[1]]. Studies have shown that the majority of women who undergo unsafe abortion are young and unmarried, poorly educated and of low socio-economic status [[2]]. An estimated 2–4.4 million adolescents resort to abortion worldwide annually [[3]]. The World Health Organization (WHO) estimates of unsafe abortion reveal that it is only in the African region that population between 15 and 24 years account for more than 50% of all abortion related mortalities [[4]]. Poverty is considered a key determinant which affects women’s decision making about unintended pregnancy as well as their access to safe post abortion care (PAC). Many women choose to terminate a pregnancy because of economic hardship and their inability to support a child [[5]].

Nigeria is on the verge of not meeting the Millennium Development Goals (MDGs) on maternal health^a^ due to a high maternal mortality ratio in the country estimated to be 630 maternal deaths per 100,000 live births. Recent evidence has shown that a major factor in this trend is the high incidence of abortion in the country [[6]]. Despite the fact that abortion is considered illegal in Nigeria, it is still widespread. The 2003 Nigeria Demographic and Health Survey (NDHS) estimated that 12% of pregnancies in Nigeria end in abortion [[7]]. A 12 year review of women who had undergone induced abortion in Ile-Ife in Nigeria showed that 30% of the cases of mortality were aged between 15–20 years and 37% were students [[8]].

In most parts of the world, abortion has declined probably due to the fact that more women are using contraceptives. Nigeria currently has a high fertility rate, coupled with low family planning practice, as well as relatively poor access to healthcare. Family planning usage is estimated to be around 15 per cent in Nigeria [[9]]. The high unmet needs^b^ for contraception lead to high number of unwanted pregnancies hence the demand for abortion. The 2008 NDHS revealed that about 20% of married women have an unmet need for family planning which comprises 15% for spacing and 5% for limiting.

Henshaw [[10]] found that an estimated $19 million are spent annually in Nigeria in treating unsafe abortion complications, and that it would cost only $4.8 million to prevent those unintended pregnancies, a cost-benefit ratio of four to one. Further, official statistics do not include privately performed abortions, only a small proportion of which are detected when women with obstetrical complications due to unprofessional procedures apply to hospitals [[11]].

In Nigeria, unsafe abortion has been described as a major issue, especially where 80 percent of patients admitted to hospitals with unsafe abortion-related complications are adolescent girls [[12]]. A host of complications are associated with unsafe abortion and studies have shown morbidity as high as 40% [[13]]. Methods employed in procuring abortion range from ingestion of toxic substances, alcohol mixed with potash or analgesic overdose to surgically induced methods by a variety of untrained individuals [[14]].

In Nigeria, abortion is permitted only in instances where it is necessary to save the life of the woman and an estimated 610,000 of such abortions are performed each year [[15]]. Many of these are performed unsafely and evidence from hospital-based studies in the country have revealed that 25 abortions occur per thousand women in the reproductive age group [[16]]. A host of complications are associated with unsafe abortions including haemorrhage, infection, uterine perforation, genital laceration, tetanus and infertility.

Empirical evidence on the demand for abortion and post abortion care has received less attention in the literature. Available studies have showed various socio-economic factors that determine the demand for abortion and post abortion care. For instance, Agadjanian [[17]] showed in the case of Mozambique, that abortion was prevalent among women under 30 years of age (74%), unmarried (58%) and still in school (36%). Also, Powell-Griner and Trend [[18]] found that women are more likely to obtain abortions if they have one or more of the following characteristics: residence in metropolitan areas, no previous children, and completion of high school, black, and unmarried.

Other studies showed that the cost of travel, price of abortion and urbanization had negative effects on abortion demand while religion (percentage of Catholics) positively influenced the demand for abortion [[19]]. Medoff [[20]] found average income, labour force participation rate and average price of abortion to be positively related to abortion demand. Madoff [[21]] also found that abortion ratios and abortion rates of unintended pregnancies were more sensitive to increase in abortion price than when both unintended and intended pregnancies are analyzed together. The availability of abortion services was also found, by some researchers, to be significant in the decision to abort [[22]–[25]].

The purpose of this study was to identify the determinants of abortion and post abortion care in the context of Nigeria with emphasis on Ibadan city. Such evidence on the magnitude and structure of the demand for abortion is critical for policymakers, providers and advocates seeking to mobilize resources to improve the situation.

## Methods

### Model specification

The model adopted in this study follows King, Myers and Byrnes [[26]]. Similar specifications have also been employed in the literature by various researchers [[19], [20], [27]–[29]]. The model was developed from the economic theory of demand. The decision to abort was modelled as a function of the full price of abortion and the respondent’s characteristics. This is based on the assumption that abortion is a posterior decision (the decision by a pregnant woman not to have the child). The demand for abortions is modelled in terms of the explicit costs at the time of the abortion decision [[28]]. The abortion choice (given a pregnancy) is thus binary in nature as the effect of variables on the decision to abort is the negative of the decision to keep the pregnancy.

A probit model is used to estimate the likelihood that a pregnancy will be aborted. It is assumed that when pregnant, two choices of whether to progress with or terminate the pregnancy are presented to a pregnant woman and one of the alternatives must be chosen. It is posited that the maximum utility that a woman receives or expects to receive by making either choice can be expressed as functions of characteristics specific to the individual. Define the indirect utility received by the already pregnant *ith* individual choosing alternative A as

(1) $$U_{\mathrm{iA}}=\sum_{j=1,k}\beta_{\mathrm{jA}}X_{\mathrm{ij}}+u_{\mathrm{iA}}$$

Where *A* is either choice 0 (birth) or 1 (abortion), *j* is an index representing 1 through *k* variables, and *u*_*iA*_ is a random error term associated with each equation. There exists an unobservable (latent) random variable of the difference in utilities such that two equations indicated by (1) can be expressed as

(2) $${y_{i}}^{*}=U_{i1}-U_{i0}=w_{i}+u_{i}=\sum_{j=1,k}\beta_{j}X_{\mathrm{ij}}+u_{i}$$

Where *β*_*j*_ *= β*_*j1*_*- β*_*j0*_*, u*_*i*_ *= u*_*i1*_*- u*_*i0*_*and X*_*i1*_ *= 1*. However, there exist a limitation to observations on the outcomes of the decision-making process where

(3) $$y_{i}={\{}_{0,\mathrm{otherwise}}^{1\mathrm{if}u_{i1}>u_{i0}\mathrm{or}u_{i}>-w_{i}}$$

If the disturbance in (1) is assumed to be independently and identically distributed (normal distribution) then a probit model can be specified for maximum likelihood estimation of the parameters β_j_^1^, the probability that a woman chooses to abort, P_i_, is

(4) $$P_{i}=\mathrm{Prob}\;\left(y_{i}=1\right)=\mathrm{Prob}\;\left(u_{i}>-w_{i}\right)=\;F\;\left(w_{i}\right)$$

Where F ( ) represents the normal cumulative distribution function.

### Empirical models

For the purposes of estimation, the reduced form equation can be presented as follows;

(5) Abi=αi+β1lnIncomei+β2Seduci+β3Meduci+β4Feduci+β5Mstati+β6Agei+β7AgeSqi+β8Religiosityi+εi

(6) PACi=αi+β1lnIncomei+β2Seduci+β3Meduci+β4Feduci+β5Mstati+β6Agei+β7AgeSqi+β8Religiosityi+εi

Equation (5) estimates the socio-economic variables that influence the demand for abortion. The model includes socio-economic variables such as income, age, education, marital status and religious commitments. The second model specified in equation (6) estimates the effects of socio-economic variables on post abortion care demand. A detailed explanation of each of the variables is presented in Table 1.

**Table 1:** Variable description

**Variables**	**Description**
*Ab*	Dummy for abortion which takes the value of 1 if a woman aborted a baby and 0 otherwise
*PAC*	Dummy for post abortion care which takes the value of 1 if the respondent sought post abortion care and 0 otherwise
*lnIncome*	Log of total income of respondent
*Seduc*	Dummy for education of respondent which takes the value of 1 if attained any formal level of educated and 0 otherwise
*Meduc*	Dummy for mother's education which takes the value of 1 if attained any formal level of educated and 0 otherwise
*Feduc*	Dummy for father's education which takes the value of 1 if attained any formal level of educated and 0 otherwise
*Mstat*	Dummy for marital status of respondents which takes the value of 1 if currently married and 0 otherwise
*Age*	Actual age of the respondents reported in years
*Agesq*	Square of respondent's age
*Religiosity*	A categorical variable capturing the respondents’ engagement in religious activities. The respondents were asked to chose between three options: Not at all, infrequent and frequent

### Data

Data used for the analysis was sourced from a primary hospital-based survey of women of reproductive age. Women between the ages of 19–49 years attending antenatal, postnatal and women in the outpatient ward were selected using a purposive sampling method. Adolescents below age 19 were excluded due to the fact that they are considered to be relatively young and sexually inactive according to NDHS 2008. The women completed a self-administered questionnaire that contained questions regarding the social-demographic characteristics, sexual behavior, demand for abortion, the cost of abortion and post abortion care. The questionnaires were completed by the women. This was to allow the women to freely respond to the questionnaire without feeling embarrassed.

The co-operation of medical officers at these institutions was solicited to assist in the administration of the questionnaires to the women in the sample. While the respondents were allowed to complete the questionnaires independently, the medical officers provided guidance/assistance when required. Allowing medical officers to assist respondents was to increase the confidence of respondents in providing such sensitive information as medical officers are more likely to be trusted than ordinary individuals. Respondents were further assured of confidentiality and the fact that information obtained would be used for research purposes only. The medical officers were not given any special training in data collection procedure but were guided on proper filling of the questionnaires. A senior doctor assisted in vetting the completed questionnaires. 501 questionnaires were planned to be administered but due to time constraint only 384 questionnaires were eventually administered.

### Ethical consideration

Given the nature of the study, the sampling and data collection procedure ensured confidentiality. In addition, the respondents were required to sign informed consent form after the objectives and procedures of the study had been explained to them. Although there was no immediate gain in terms of incentives for the participants, they were made to understand that their participation in the study will contribute towards future policy making and assist in the design of programmes to help females (young adults) with the problem of unwanted pregnancy. The respondents were also informed about the possibility for them to decline to participate in the study.

In addition to the above procedure, the study obtained ethical clearance from the University College Hospital Research Ethics Review Committee, University of Ibadan, Nigeria.

## Results

### Descriptive statistics

Table 2 presents the mean and percentages of the variables included in the study. Table 2 shows that about 191 (62%) out of the 308 respondents demanded for abortion. Approximately 100 (52.3%) out of the 191 women who demanded abortion also demanded for post abortion care over the period of study. About 108 (35.1%) of respondents were employed in private institutions. Government employees were 92 (29.2%) out of the sample. While 25 (8.1%) respondents worked as housewives, 83 (26.9%) of the respondents were unemployed. The average age of the respondents was 28 years. Majority of the respondents were single (46.8%) while about 39.0% were married. A relatively smaller percentage of the population was divorced (10.4%), separated (1.9%) and widowed (1.9%). About 184 (59.7%) of the respondents were from the Yoruba tribe while 72 (23.4%) and 24 (7.8%) were from the Igbo and Hausa tribes respectfully. All other tribes made up 28 (7.9%) of the total respondents. Average income across respondents was 56, 109.48^c^ Nigerian Naira (₦). While this may be higher than the typical income of a woman in Ibadan, it may be explained by the large number of women with higher education included in the sample.

**Table 2:** Descriptive statistics

**Variable**	**Observations**	**Mean**	**Number (%)**
Demand for abortion (yes)	308		191(62%)
**Occupation**			
Housewife	308		25 (8.1)
Government	308		92 (29.9)
Private	308		108 (35.1)
Unemployed	308		83 (26.9)
**Age (years)**	308	28	
**Age range**			
19–24	308		71 (23.1)
25–30	308		139 (45.1)
31–49	308		98 (31.8)
**Marital status**			
Single	308		144 (46.8)
Married	308		120 (39.0)
Divorced	308		32 (10.4)
Widowed	308		6 (1.9)
Seperated	308		6 (1.9)
**Religiosity**			
not at all	308		12 (3.9)
Infrequent	308		150 (48.7)
Frequent	308		146 (47.4)
**Tribe**			
Yoruba	308		184 (59.7)
Ibgo	308		72 (23.4)
Hausa	308		24 (7.8)
Others	308		28 (9.1)
**Income**	308	₦ 56,109.48	
**Denomination**			
Catholic	308		91 (29.5)
Islam	308		73 (23.7)
Protestant	308		103 (33.4)
Orthodox	308		20 (6.5)
Traditional	308		21 (6.8)
**Post abortion care (Yes)**	191		100 (52.3)
**Respondents' education**			
None	308		15 (4.9)
Primary	308		9 (2.9)
Secondary	308		40 (13.0)
Post secondary	308		244 (79.2)
**Mother’s education**			
None	308		98 (31.8)
Primary	308		53 (17.2)
Secondary	308		61 (19.8)
Post secondary	308		95 (30.8)
**Father’s education**			
None	308		67 (21.8)
Primary	308		42 (13.6)
Secondary	308		60 (19.5)
Post secondary	308		138 (44.8)

### Determinants of abortion demand

Table 3 shows the results from two different models on the socio-economic determinants of abortion demand. The first model includes respondent’s income, education, age, marital status and parent’s education, while the second model introduces cost of abortion. The education variables were automatically dropped when the total cost of abortion was introduced in the second model. Results from the first model shows that income, age, marital status and mother’s education were significant determinants of abortion demand.

**Table 3:** Probit model of socio-economic determinants of abortion demand

	**Model 1**	**Marginal effects**	**Model 2**	**Marginal effects**
**Respondent**				
Education	−0.43762	−0.16811		
	(−0.38399)	(−0.13831)		
Marital status	−0.29859*	−0.11866*	0.57205	0.01114
	(−0.17013)	(−0.06715)	(−0.47767)	(−0.01207)
Log income	0.29933***	0.11927***	0.19468	0.00418
	(−0.08795)	(−0.03502)	(−0.24015)	(−0.00579)
Age squared	−0.00152	−0.0006	0.00902**	0.00019*
	(−0.00111)	(−0.00044)	(−0.00458)	(−0.0001)
Age	0.12404*	0.04943*	−0.51442**	−0.01103*
	(−0.07456)	(−0.02971)	(−0.23947)	(−0.00583)
Log total cost of abortion			0.08951	0.00192
			(−0.18315)	(−0.004)
**Religiosity**				
Infrequent			−3.58213***	−0.25427*
			(−0.56451)	(−0.14855)
Frequent			−4.44578***	−0.51820***
			(−0.45017)	(−0.15189)
**Mother's education**	−0.38239*	−0.15060*		
	(−0.2314)	(−0.08929)		
**Father's education**	−0.01178	−0.00469		
	(−0.27146)	(−0.10813)		
Constant	−4.60410***		10.02630***	
	(−1.38469)		(−3.32549)	

**Note:** ***significant at 1%; **significant at 5%; *significant at 10%.

**(1)** is model without total cost of abortion demand.

**(2)** is model with log of total cost of abortion demand.

Robust standard errors are reported in parenthesis.

The relationship suggests that women with higher income were more likely to demand for abortion. That is, as the level of income increases, the probability of demanding for abortion also increases. This relationship was significant at 1% with a marginal effect of 0.1. However, in the second model where the total cost of abortion was introduced, both income and the cost of abortion were not significant determinants of abortion demand.

The results also showed, from the first model, that married women were less likely to demand for abortion, relative to women who were not married. Similarly the age of the respondent showed a positive relationship with the probability of abortion demand in the first model. However, when cost of abortion was introduced in the model, age showed negative and statistically significant (at 5% level) relationship with abortion demand.

Both the respondent’s and father’s education were not significant but the mother’s education showed negative and significant relationship with abortion demand. Women with educated mothers were less likely to demand for abortion with a marginal effect of approximately −0.15.

The respondent's religious commitment showed negative and significant relationship at 1%. The variable measures religious inclination of respondent and has three categories namely: Not at all, infrequent and frequent engagement in religious activities. The results suggest that more religious women were less likely to demand abortion, relative to women who were not religious at all. This relationship was strongly significant irrespective of whether religious participation was frequent or infrequent.

### Determinants of post abortion care

Table 4 shows that post abortion care demand was only influenced by income, age and marital status of the respondent. Models four (4) and five (5) are distinguished by the introduction of the cost of abortion in model five. The results in both models indicate that women with higher income levels were more likely to demand for post abortion care relative to women with lower income levels. While respondent's age was not significant in model 4, the relationship was significant when cost of abortion was introduced in model 5. The result showed that older women were less likely to demand post abortion care. Similar to the findings in the demand for abortion model, religiosity was a significant determinant of post abortion care. Relative to those who did not engage in any religious activity, women who frequently or infrequently participated in religious activities were less likely to demand post abortion care.

**Table 4:** Probit model of socio-economic determinants of post abortion care

**Variables**	**Model 4**	**Marginal effects**	**Model 5**	**Marginal effects**
**Respondent**				
Education	−0.30437	−0.11781		
	(−0.36524)	(−0.13596)		
Marital status	−0.31512*	−0.12498*	−0.20449	−0.00798
	(−0.1682)	(−0.0663)	(−0.39562)	(−0.01687)
Log income	0.29524***	0.11727***	0.45440*	0.01673
	(−0.08851)	(−0.03511)	(−0.27108)	(−0.0111)
Age square	−0.00121	−0.00048	0.00474**	0.00017*
	(−0.00109)	(−0.00043)	(−0.00219)	(−0.0001)
Age	0.09984	0.03965	−0.31265*	−0.01151
	(−0.0738)	(−0.02932)	(−0.16698)	(−0.00727)
Log total cost of post abortion care			0.20231	0.00745
			(−0.22262)	(−0.00815)
Religion1			−4.04989***	−0.42199***
			(−0.47173)	(−0.1375)
Religion2			−4.48105***	−0.61794***
			(−0.37773)	(−0.12269)
Mother's education	−0.13729	−0.05435		
	(−0.22705)	(−0.08949)		
Father's education	−0.07334	−0.02906		
	(−0.26551)	(−0.10493)		
Constant	−4.32392***		4.50871	
	(−1.38238)		(−3.38419)	

**Note:** ***significant at 1%; **significant at 5%; *significant at 10%.

**(4)** is model without total cost of post abortion care demand.

**(5)** is model with log of total cost of post abortion care demand.

Robust standard errors are reported in parenthesis.

Further, the marital status of respondents showed negative and significant relationship with post abortion care demand. That is, married women were less likely to report for post abortion care relative to unmarried women. Women’s education level and parents’ education did not show any statistically significant relationship with post abortion care demand. Total cost of post abortion care did not also show any statistical significance.

## Discussion

The findings of the study suggest that income of women play an important role in the demand for both abortion and post abortion care. This means that women with relatively lower income levels are constrained from going for abortion if they wish to terminate their pregnancies. This findings is understandable in the case of Ibadan for two reasons; first, public health care insurance is generally underdeveloped in the Ibadan metropolis and Nigeria as a whole. Hence income still plays a crucial part in any form of health care service utilization. Secondly, abortion services are considered illegal in the metropolis, like in many other places in Nigeria, with several risks involved such as the death of patients, hemorrhage, uterine perforation, genital laceration, tetanus and infertility hence the cost involved in providing such service is high.

The constraints posed by income in the demand for abortion and post abortion care may have some positive implications. That is, fewer women are likely to demand abortion services due to this constraint. While this may aid the fight against abortion in the metropolis, there is need to ensure that it does not lead to increase in self-induced abortion cases. This situation is much more critical when complications occur and post abortion care is required. Women who have undergone abortion are safer when they stay under the care of qualified health care professionals for observation and appropriate treatment.

This finding conforms with that of Medoff [[20]] who found that income of women has a significant positive impact on the demand for abortion services in Columbia. Medoff [[20]] also highlighted the importance of income in the demand for abortion and post abortion care by showing that women who do not directly pay for abortion related services are more likely to demand for abortion [[21]].

The results also suggest that marital status significantly influence the demand for abortion and post abortion care. In both cases, married women are less likely to go for abortion or post abortion care. This is expected as married women are generally more likely to keep their pregnancies till birth, except in cases where medical conditions require that an abortion is performed to save the life of the mother. Further in cases where married women decide to abort, they are more likely to continue to seek for treatment after the abortion to ensure that they do not have complications or when they do they are treated appropriately. This finding is similar to the findings of Powell-Griner [[18]] that women in the United States of America who are unmarried are more likely to abort their babies than married women.

The finding on age was expected as younger women were expected to be more likely to abort. This is because teenage pregnancy and pregnancy among young women in general are not acceptable in Nigerian communities, including the Ibadan metropolis. Young women in this condition face several stigmas from society, drop out of school and in some cases are rejected by their families. To avoid these embarrassments and challenges, young women who become pregnant are likely to easily opt for abortion. The negative relationship estimated from this study may be mitigated by the increasing public education against abortion among young women in Nigeria. The purpose of such education by government and other non-government organizations (NGOs) is to discourage abortion among young women due to the high risks involved.

Interestingly, women education did not show any statistically significant relationship with both abortion and post abortion care demand. This contradicts the findings of Powell-Griner [[18]] that women who had completed high school were more likely to abort. It is worth mentioning that, while education and income are usually correlated and both were expected to be significant in determining abortion and post abortion care demand, only income was found to be significant. This may be explained by the weight given to each of these variables in making the decision to abort. The level of income does not only influence the willingness to abort but also the ability to purchase such service. Education attainment may only, to a limited extent, influence the willingness to demand abortion services.

However, mother’s education was a significant negative determinant of abortion care demand. This implies that women whose mothers had some education were less likely to abort relative to women whose mothers had no formal education. While a similar relationship was found for father’s education, the relationship was not significant. The results suggest that mothers have greater influence on the decisions of their daughters than the father.

The findings also suggest that religiosity of respondents plays an important part in the demand for abortion and post abortion care. That is, women who frequently or infrequently engage in religious activities are less likely to demand abortion or post abortion care. The relationship was expected as most religious denominations in Ibadan (and Nigeria as a whole) frown at abortion. The moral values taught by religious organizations may also explain the significant negative relationship established.

Surprisingly, the cost of abortion and post abortion care did not show any statistical significance even though they positively influenced demand for abortion and post abortion care. Medoff [[20]] and Deyak and Smith [[19]] found similar relationships in their respective studies.

## Conclusion

The study sought to estimate a model of the demand for abortion and post abortion care. The study used primary data collected from the Ibadan metropolis of Nigeria and a probit model to determine the factors that influence the demand for abortion and post abortion care.

The findings suggest that women’s income, age, marital status and mother’s education were significant determinants of abortion demand. The cost of abortion and women’s education did not show any statistical significance. Similarly, women’s income, age and marital status were significant variables in the demand for post abortion care.

The findings imply that government policies should be directed towards effective campaign to reduce unwanted pregnancies and unnecessary abortion. Abortion is not only associated to the poor as the higher income earners were more likely to demand abortion. Policies should therefore target both the poor and rich in reducing abortion demand and encouraging post abortion care demand as the later could be debilitating. Further, there is need to focus policies to control abortion on both the young and old in society. This will be important in reducing the prevalence of abortion as well as reducing abortion related mortalities and disabilities.

While the findings of this study may be relevant for abortion related policies, the extent to which the findings can be generalised may be limited as the scope was restricted to the Ibadan metropolis due to financial and time constraints. Future studies should attempt broadening the scope to cover other states in Nigeria.

## Endnotes

^a^MDG on maternal health was to reduce maternal mortality by three quarters between 1990 and 2015.

^b^Unmet need for family planning or contraception is defined as the percentage of married women who want to space their next birth or stop child bearing entirely but are not using contraception (NDHS, 2008).

^c^Approximately US$ 374 at an exchange rate of US$1 = ₦150.

## Competing interests

The authors declare they have no competing interests.

## Authors’ contributions

BOA conceived the study and undertook the data collection, JN analysed the data and wrote the manuscript. Both authors read and approved the final manuscript.
